# Sustained Remission in Patients with Primary Immune Thrombocytopenia after Romiplostim Tapering and Discontinuation: A Case Series in Real Life Management in Spain

**DOI:** 10.1155/2017/4109605

**Published:** 2017-06-11

**Authors:** María-Eva Mingot-Castellano, Carlos Grande-García, David Valcárcel-Ferreiras, Clara Conill-Cortés, Loreto de Olivar-Oliver

**Affiliations:** ^1^Hematology Department, Hospital Regional Universitario de Málaga, Av. de Carlos Haya, s/n, 29010 Málaga, Spain; ^2^Hematology Department, Hospital Universitario 12 de Octubre, Avda. Córdoba, s/n, 28041 Madrid, Spain; ^3^Hematology Department, Hospital Universitari Vall d'Hebron, Passeig de la Vall d'Hebron 119-129, 08035 Barcelona, Spain; ^4^Amgen S.A., Moll de Barcelona, Edifici Sud, Planta 7, 08039 Barcelona, Spain

## Abstract

Romiplostim, a thrombopoietin-receptor agonist (TPO-ra), is a highly effective option in primary immune thrombocytopenia (ITP), with 80–90% of patients achieving platelet responses after few weeks of treatment. The evidence showing remissions, that is, sustained platelet counts after romiplostim discontinuation, in patients with ITP refractory to immunosuppressive therapy is steadily increasing. However, there is a lack of guidelines or recommendations addressing how and when to taper romiplostim in clinical practice in patients maintaining elevated and stable platelet counts. Furthermore, given the high heterogeneity of ITP patients, no associated predictive factors have been currently identified. Here, we present 4 representative clinical cases of the daily clinical practice in Spain comprising newly diagnosed, persistent, and both splenectomized and nonsplenectomized chronic ITP patients treated with romiplostim, achieving and maintaining clinical remission (platelet count ≥ 50 × 10^9^/L for 24 consecutive weeks in the absence of any treatment for ITP) after treatment tapering and discontinuation, without observed safety concerns. Prospective studies identifying clinical and biological predictive factors of sustained response are warranted.

## 1. Introduction

Primary immune thrombocytopenia (ITP) is an autoimmune disease characterized by low platelets counts resulting in an increased risk of bleeding [1, 2]. The pathogenesis of this chronic disorder is thought to be caused due to both platelet destruction and suboptimal platelet production [3, 4].

Classically, treatments focused on reducing platelet destruction in the short term (intravenous immunoglobulins, steroids, and anti-D immunoglobulin) or in the long term (rituximab and splenectomy) but failed to achieve or maintain an endurable response in certain patients and were associated with severe adverse events [5–10]. More recently, a novel generation of treatments, the thrombopoietin-receptor agonists (TPO-ras), has been developed aiming to stimulate megakaryocyte growth and increase platelets production [11, 12].

Romiplostim, a TPO-ra that interacts with the extracellular domain of the thrombopoietin receptor, has demonstrated rapid and sustained platelet increases in approximately 85% of both splenectomized and nonsplenectomized ITP patients, while reducing the use of concomitant medications, the requirement of splenectomy as salvage therapy, and, more importantly, the incidence of bleeding [13–17].

While the majority of patients require long-term TPO-ra treatment to maintain platelet responses, there is increasing evidence showing that certain patients may achieve prolonged remission after TPO-ra discontinuation [18–31]. Despite this, currently there are no unified and validated criteria or guidelines regarding how and when to taper and discontinue TPO-ra treatment in responders or a characterization of the patients that may be benefitted from this practice.

Here we report a small series of clinical cases of ITP patients refractory to immunosuppressive therapy who were treated with romiplostim. After achieving sustained responses, romiplostim was tapered and finally discontinued without relapsing occurrence to date. Our objective is to describe representative experiences of the management of these ITP patients in daily clinical practice.

## 2. Case Presentation

### 2.1. Case 1: Newly Diagnosed ITP

A 48-year-old man with a controlled thyroid nodule and tobacco and cannabis abuse presented with ecchymosis in his limbs by August 2014. The laboratory test found isolated thrombocytopenia (platelet count 9 × 10^9^/L) without atypical features. The bone marrow examination (aspiration and biopsy, April 2015) revealed results compatible with ITP.

**Figure 1:**
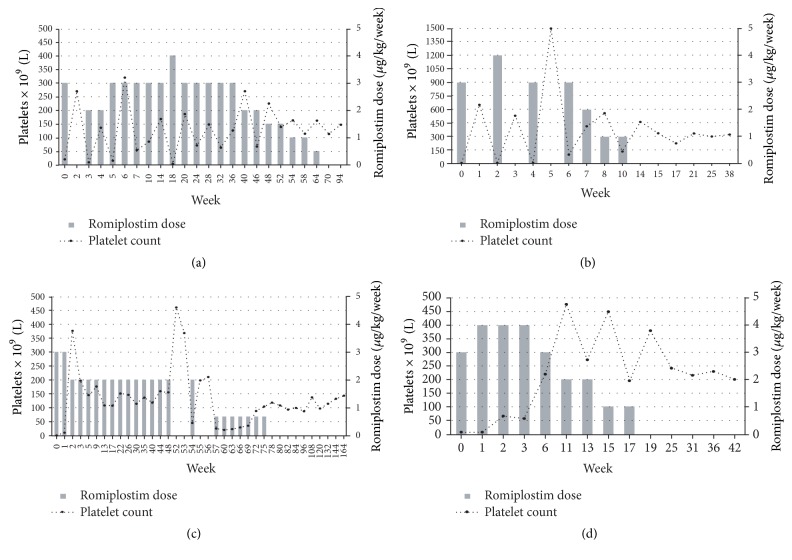
Platelet count and romiplostim dose evolution: (a) case 1: newly diagnosed ITP; (b) case 2: persistent ITP; (c) case 3: chronic ITP; splenectomized; (d) case 4: chronic ITP; nonsplenectomized.

He started prednisone (1 mg/Kg, daily). After 3 weeks of treatment, there was no response (platelet count 12 × 10^9^/L). Aiming to perform the thyroid nodule extirpation, immunoglobulins were also administered (1 g/Kg daily for 2 days). After 5 weeks with no further response (platelet count 20 × 10^9^/L), prednisone was tapered and romiplostim was started at 3 *µ*g/Kg, observing a rapid response after two weeks (platelets 270 × 10^9^/L). Steroids were discontinued and romiplostim was discontinued for one week, observing a new platelet decrease to 9 × 10^9^/L after which romiplostim was rescheduled at 2 *µ*g/Kg dose, achieving a stable platelet response and allowing the thyroid surgery (October 2014, follicular adenoma, nonmalignant). Romiplostim was maintained at 3 *µ*g/Kg with stable platelet counts (Figure 1(a)) until January 2015, when there was a platelet decrease (3 × 10^9^/L) without hemorrhagic signs, managing titrating romiplostim to 4 *µ*g/Kg.* Helicobacter pylori* was then eradicated after positive breath test.

Romiplostim continued at 3 *µ*g/Kg thereafter with acceptable platelet counts (30–150 × 10^9^/L) and was initially tapered to 2 *µ*g/Kg (month 9 after diagnosis; platelet count: 270 × 10^9^/L) and further decreased by 0.5 *µ*g/Kg every two months while maintaining platelet response (Figure 1(a)). The treatment was finally discontinued 16 months after ITP diagnosis, achieving its current state of remission by June 2016 (platelet count ≥ 50 × 10^9^/L for 24 consecutive weeks in the absence of any treatment for ITP). No adverse events were observed.

### 2.2. Case 2: Persistent ITP

A 64-year-old male was assisted in emergency department because of melena, mild hemorrhagic lesions in thorax and abdomen, asthenia, and progressive adynamia in April 2015. He had a history of tobacco abuse, ischemic stroke, and arterial hypertension and was receiving acetylsalicylic acid, atorvastatin, rizatriptan, ferrous sulfate, and losartan-hydrochlorothiazide. The blood count showed hemoglobin 7.9 g/dL and platelet count 3 × 10^9^/L. The diagnosis was ITP after excluding other possibilities including bone morrow study. A digestive endoscopy revealed hiatus hernia, Schatzki ring, and severe erythematous gastritis in body and fundus. Positive results for* Helicobacter pylori* in stool test were obtained.

After diagnosis, aspirin was stopped, and* H. pylori* eradication treatment and immunoglobulins (1 g/Kg daily for 2 days) were started. After 4 days without response (platelet count: 10 × 10^9^/L), the patient initiated dexamethasone 40 mg daily for 4 days (without tranexamic acid) achieving 410 × 10^9^/L platelets. Fourteen days after ITP diagnosis, the* H. pylori* test was negative and the platelet count decreased to 2 × 10^9^/L. Five additional cycles of dexamethasone were administered (40 mg daily for 4 days every 15 days), not maintaining a stable response (platelet count: 1–10 × 10^9^/L before dexamethasone cycles; 187–410 × 10^9^/L one week after). During the fifth cycle, the patient was then hospitalized after blood transfusion due to digestive bleeding with hemodynamic angina.

Romiplostim treatment was then started at 3 *µ*g/Kg. High platelets response was observed after one week (platelets 649 × 10^9^/L). Platelet count decreased to 2 × 10^9^/L after 1 week without romiplostim being administered. It was reinitiated at 4 *µ*g/Kg since the first platelet response was assumed to be caused by the combination of steroids and romiplostim. A new increase in platelet count (528 × 10^9^/L) was observed. After 6 weeks of intermittent administration, the platelet count remained stable at 2 *µ*g/Kg (Figure 1(b)), and the aspirin treatment was restored. Romiplostim intervals of administration were sequentially spaced and the treatment was finally discontinued after 14 weeks of romiplostim onset, achieving its current state of remission in May 2016. No new safety signals were observed.

### 2.3. Case 3: Chronic ITP: Splenectomized

A 21-year-old male was diagnosed of ITP in August 2008 after presenting sustained severe mucocutaneous manifestations (age at diagnosis: 13 years old). At diagnosis, the blood count showed platelet count 5 × 10^9^/L and hemoglobin 11.2 g/dL with iron deficiency and negative direct Coombs test. After being treated with immunoglobulins and corticosteroids achieving limited response, the patient was splenectomized in July 2009 followed by a complete response, sustained for 30 months. After relapsing (severe asthenia, mucocutaneous manifestations, platelet count 12 × 10^9^/L, and hemoglobin 11 g/dL), he refused to be treated with steroid and immunoglobulin treatment was indicated (1 g/Kg daily for 2 days) with short-term response (platelet count 296 × 10^9^/L and 12 × 10^9^/L after 4 and 10 days, resp.), followed by a second cycle (0.8 g/Kg single dose) 21 days thereafter achieving a similar response (platelet count 134 × 10^9^/L and 3 × 10^9^/L after 4 and 10 days, resp.) with presence of petechiae and ecchymosis. The patient was then treated with four 15-day cycles of dexamethasone (40 mg daily for 4 days) in monotherapy without observing further response (platelet count 9 × 10^9^/L, without bleeding).

As he wanted to continue with an active life, at June 2013 (18 year-old; platelet count 2 × 10^9^/L) he started romiplostim 3 *µ*g/Kg, observing an increase in platelet count after 2 weeks to 378 × 10^9^/L. The patient maintained stable values of platelets between 109 and 178 × 10^9^/L for 12 months with a romiplostim dose of 2 *µ*g/Kg (Figure 1(c)). At the 12-month visit romiplostim was interrupted for 2 weeks, followed by a decrease in platelet count observed in the third week without treatment (47 × 10^9^/L). After romiplostim retreatment (2 *µ*g/Kg every 3 weeks) the number of platelets steadily increased (Figure 1(c)) and treatment was discontinued 18 months after romiplostim onset. After 20 months of treatment withdrawal the patient is still under clinical remission, with no safety signals observed.

### 2.4. Case 4: Chronic ITP: Nonsplenectomized

A 67-year-old woman with history of hypercholesterolemia, cholecystectomy, and appendectomy was diagnosed with ITP in 2000. A bone marrow aspiration and biopsy were performed with results compatible with ITP. The patient was initially treated with prednisone (1 mg/kg), achieving a sustained complete response until 2006 when she relapsed presenting concomitant hemolytic anemia (Evans syndrome) unresponsive to corticoids. She regained full response after four weekly cycles of rituximab (375 mg/m^2^), maintaining adequate platelet counts (>100 × 10^9^/L) during 9 years.

In July 2015, the patient presented a new episode of severe thrombocytopenia (platelet count 3 × 10^9^/L) and epistaxis, starting biweekly dexamethasone (40 mg daily for 4 days). After 3 cycles without observing clinical response, eltrombopag was initiated at 50 mg/day and rapidly increased to 75 mg/day for 3 weeks. The platelet count remained low (6 × 10^9^/L) and, in September 2015, the TPO-ra treatment was switched to romiplostim 3 *µ*g/Kg, observing a reduction in cutaneous bleeding diathesis, but without platelet count increases after one week. Romiplostim was then titrated to 4 *µ*g/Kg, with a discrete increase in platelet count observed after 2 weeks, achieving 219 × 10^9^/L after 5 weeks. The dose was sequentially tapered to 3 *µ*g/Kg, to 2 *µ*g/Kg in alternate weeks one month thereafter, and to 1 *µ*g/Kg in alternate weeks by December 2015 (Figure 1(d)). Romiplostim was finally discontinued in January 2016 (platelet count 379 × 10^9^/L), achieving and maintaining remission with no new safety signals observed.

## 3. Discussion

Given the lack of published guidelines or recommendations addressing the management of patients with ITP treated with TPO-ra that achieve sustained platelet counts, reporting and sharing real-world clinical experiences are paramount. Here we report four clinical cases of adult patients with primary ITP at different stages (newly diagnosed [diagnosis to 3 months], persistent [3–12 months' duration], and chronic [>12 months' duration]) treated with romiplostim who achieved clinical remission, maintaining sustained platelet counts after its discontinuation (Table 1). All 4 patients had failed to multiple previous therapies but differ in the previous response. Two patients (case 1 and case 2) achieved limited/no response to previous treatments, including steroids and immunoglobulins, whereas one patient (case 3) completely responded to splenectomy, and one patient (case 4) achieved complete response to prednisone and rituximab, but not to another TPO-ra (eltrombopag). As previously observed in several trials [13–15], patients presented a very good tolerance to romiplostim, even in cases 2 and 4, which had several comorbidities that hamper their management with first-line treatments such as steroids.

Several other reports have demonstrated sustained remission and a positive safety profile after romiplostim discontinuation, mainly in chronic relapsing or refractory ITP patients. A multicenter French observational study [21] including 28 out of 54 patients with complete response after TPO-ra treatment reported 8 (15%) patients remaining in sustained remission off-therapy (median follow-up after treatment discontinuation: 13,5 months; range: 5–27). Červinek et al. [24] published data of 11 out of 46 relapsed or refractory ITP patients under remission after discontinuing TPO-ra, with a median follow-up of 33 months (range: 16–54), without side-effects observed in the long term. Santoro et al. [25] studied 39 ITP patients treated with TPO-ra after failure to one or more therapy lines observing a 13% (*n* = 5) of persistent responders off-treatment, with a median follow-up of 2.3 years. These results have been recently reproduced in a bone marrow study [31] including 169 patients with ITP, of which 24 (14%) achieved remission after a median of 52 weeks (range 6–124) of second-line romiplostim treatment. The median duration of remission during the study was 88 weeks (range 29–154), with 21 of the 24 patients still in remission at the last observation on study. A post hoc analysis [32] indicated that ITP duration ≤ 1 year could be a potential predictor for remission, although additional studies are needed to confirm this possibility. Similar results of sustained remissions after TPO-ra treatment and discontinuation have also been reported in several small case series of ITP patients [18–20, 22, 27–29, 33].

An integrated analysis of eight romiplostim ITP trials (*n* = 949) was performed by Bussel et al. [34]. Twenty-seven patients maintained platelet counts of ≥50 × 10^9^/L for ≥26 consecutive weeks without any treatment for ITP. The median time from initiation of romiplostim treatment to onset of remission was 31 weeks (range, 3.1–181 weeks) and the median duration of remission was 42 weeks (range, 26–174 weeks). The short follow-up of some of the included trials and the high platelet count required to interrupt romiplostim (>400 × 10^9^/L in most trials) may have restricted these results.

There is additional evidence regarding newly diagnosed or persistent ITP patients achieving remission after TPO-ra treatment. A prospective (12-month follow-up), single-arm, phase II trial recently published by Newland et al. [30] included 75 patients with ITP diagnosed within 6 months of romiplostim onset (median: 2.2 months) who failed to at least one first-line treatment. Twenty-four out of 75 (32%) patients achieved clinical remission (platelet counts of ≥50 × 10^9^/L for 24 consecutive weeks without any ITP treatment). Mean platelet count for the first 2 months was associated with remission. A retrospective analysis of 260 patients with primary ITP reported by González-López et al. [35] observed 80 patients who discontinued eltrombopag after achieving a complete remission (platelet count > 100 × 10^9^/L). Twenty-six out of 49 (53%) evaluable patients showed sustained response (median follow-up: 9 months; range: 6–25) after discontinuing eltrombopag, with 4 patients being categorized as persistent ITP.

Carpenedo et al. [23] reported 13/27 responders who discontinued romiplostim after a mean of 44.3 weeks on therapy with sustained response maintained for a mean of 26 months. Three and 4 patients were newly diagnosed and persistent, respectively, and their long-term responses were proposed to be spontaneous. Whether our recently diagnosed patients were spontaneous responders remains unclear. Interestingly, there is a differential characteristic between them. While case 1 started remission after 16 months of romiplostim onset, case 2 started remission after 14 weeks. However, this has not been identified as a predictive factor in the published evidence.

The mechanisms and factors of remission in patients with ITP remain unknown, although some studies suggest a restoration of immune tolerance and a decrease of inflammatory state after continuous treatment with TPO-ra through the stimulation of regulatory B and T (T-reg) lymphocytes, as measured by the suppression of autologous CD4(+) CD25(−) cells' proliferation and the reduction of interleukin-2-producing CD4(+)_cells [36, 37]. On the other hand, circulating transforming growth factor-*β*1 (TGF-*β*1) is essential to preserve T-reg functions, inducing forkhead box protein 3 (Foxp3) expression and converting T-regs Foxp3(−) to Foxp3(+) [38]. In patients treated with TPO-ra agents, TGF-*β*1 levels increase while soluble CD40-ligand (sCD40L) levels decrease [36, 39], suggesting a reduction in the overall inflammatory state and supporting the nonactivation of platelets in these patients [40]. Furthermore, in patients under TPO-ra treatment, a significant elevation in Fc*γ* receptor (Fc*γ*R) IIb and a decrease in Fc*γ*R I and IIa levels have been observed [41]. Fc*γ*R is a heterogeneous group of cell-surface glycoproteins that provide links between humoral and cellular immunity. An elevated expression of the activating Fc*γ*R I and IIa along with decreased expression of the inhibitory Fc*γ*R IIb is involved in the pathogenesis of ITP [42]. In vitro phagocytosis assays show that a shift in the balance of Fc*γ*R toward inhibitory Fc*γ*R IIb on monocytes was accompanied with a considerable decrease in monocyte/macrophage phagocytic capacity in patients with ITP treated with TPO-ra [41]. Modulation of monocyte Fc*γ*R balance by TPO-ra was also found in a murine model of ITP established by transferring splenocytes from immunized CD61 knockout mice into CD611 severe combined immunodeficient mice. Romiplostim administration in ITP mice significantly upregulated inhibitory Fc*γ*R II expression and downregulated activating Fc*γ*R I expression [41]. Further analyses are warranted of whether these two mechanisms of action of TPO-ra are interconnected or if there are additional mechanisms involved.

Despite the differential characteristics of the described patients (Table 1), platelet responses were rapidly observed after romiplostim treatment (1-2 weeks) in all cases, with lower TPO-ra doses compared with the mean doses administered in the efficacy trials [14, 16]. Several other reports had observed similar low doses in long-term remitters [18–20, 22, 24, 30], being comparatively higher in patients not achieving stable platelets counts and subsequent discontinuation [24]. However, a direct causality between administered doses and remission status was not established.

Although it is widely used in clinical practice as second- or third-line treatment in ITP, there are still no guidelines addressing the management of romiplostim in responder patients. Given its high variability, the treatment and follow-up of ITP patients should be personalized and the final judgment should be made after an exhaustive characterization of individual circumstances. In this regard, the published evidence failed to identify predictive factors of remission after TPO-ra treatment [21–23, 34, 35] besides a higher platelet count in the first two months of treatment observed by Newland et al. in patients achieving remission [30] and, potentially, an ITP duration ≤ 1 year [32].

The dose tapering described in the registration trials [13, 16] has been slightly redefined in subsequent long-term extensions [15] and is the current* standard of care* for ITP patients treated with romiplostim achieving platelet response. Generally, in clinical practice, romiplostim is withheld if platelets count is over 250–400 × 10^9^/L, with dose and intervals adjustments at the physician's discretion to maintain target platelet counts. The maintenance of stable platelet counts after treatment discontinuation was either described in the short term to mid-term [18–20] (cases 2 and 4) or in the long term [22–24] (cases 1 and 3), suggesting different physiological responses to romiplostim in ITP patients and bolstering the importance of a close follow-up and treatment titration in these patients. Functional T-regs normalization might have already been induced in these patients through TPO-ra. Accordingly, TPO-ra administration has to be adjusted, aiming to fully restore platelet counts and to achieve stable T-reg populations, establishing long-term immune tolerance. As mentioned, in clinical practice, required doses and intervals are expected to differ between patients, and the minimal effective dose to maintain adequate platelet levels should be determined individually. This adjustment to the minimum effective dose should be closely monitored, with observation periods between dose modifications noninferior to 2–4 weeks to be aware about platelets counts stability.

In summary, there is an increasing evidence of patients with ITP achieving remission after romiplostim tapering and discontinuation. This might represent a paradigm shift for these patients traditionally requiring chronical treatments. The identification of predictive associated factors in prospective long-term trials is warranted, aiming to generate unified recommendations for the treatment and management of these patients in daily clinical practice.

## Figures and Tables

**Table 1:** Characteristics of ITP patients treated with romiplostim.

Case number	ITP classification	ITP evolution^*∗*^	Baseline platelet count^*∗*^	Previous ITP treatments	Initial romiplostim dose	Time to response (platelet count)^†^	Time to romiplostim taper^‡^	Time to romiplostim discontinuation^‡^	Sustained response^§^
1	Newly diagnosed	5 weeks	20 × 10^9^/L	2 (IVIG, prednisone)	3 *µ*g/Kg	2 weeks (270 × 10^9^/L)	52 weeks	16 months	Yes
2	Persistent	13 weeks	1 × 10^9^/L	2 (IVIG, dexamethasone)	3 *µ*g/Kg	1 week (649 × 10^9^/L)	1 week	14 weeks	Yes
3	Chronic; splenectomized	58 months	2 × 10^9^/L	3 (IVIG, dexamethasone, splenectomy)	3 *µ*g/Kg	2 weeks (378 × 10^9^/L)	52 weeks	18 months	Yes
4	Chronic; nonsplenectomized	180 months	6 × 10^9^/L	4 (prednisone, rituximab, dexamethasone, eltrombopag)	3 *µ*g/Kg	2 weeks (65 × 10^9^/L)	11 weeks	19 weeks	Yes

^*∗*^At the start of romiplostim treatment. ^†^Response defined as platelet count ≥50 × 10^9^/L. ^‡^Time from romiplostim onset. ^§^Defined as platelet count ≥50 × 10^9^/L for 24 consecutive weeks in the absence of any treatment for ITP. ITP: primary immune thrombocytopenia; IVIG: intravenous immunoglobulin.
